# Amelioration of Ethanol-Induced Hepatitis by Magnesium Isoglycyrrhizinate through Inhibition of Neutrophil Cell Infiltration and Oxidative Damage

**DOI:** 10.1155/2017/3526903

**Published:** 2017-08-29

**Authors:** Yan Wang, Zhenzhen Zhang, Xia Wang, Dan Qi, Aijuan Qu, Guiqiang Wang

**Affiliations:** ^1^Department of Infectious Diseases, Peking University First Hospital, Beijing 100034, China; ^2^Department of Physiology and Pathophysiology, School of Basic Medical Sciences, Capital Medical University, Key Laboratory of Remodeling-Related Cardiovascular Diseases, Ministry of Education, Beijing 100069, China

## Abstract

Alcoholic liver disease (ALD) is a leading cause of liver-related morbidity and mortality worldwide. There is no effective treatment to prevent the disease progression. Magnesium isoglycyrrhizinate (MgIG) showed potent anti-inflammatory, antioxidant, and hepatoprotective activities and was used for treating liver diseases in Asia. In this study, we examined whether MgIG could protect mice against alcohol-induced liver injury. The newly developed chronic plus binge ethanol feeding model was used to study the role of MgIG in ALD. Serum liver enzyme levels, H&E staining, immunohistochemical staining, flow cytometric analysis, and real-time PCR were used to evaluate the liver injury and inflammation. We showed that MgIG markedly ameliorated chronic plus binge ethanol feeding liver injury, as shown by decreased serum alanine transaminase and aspartate aminotransferase levels and reduced neutrophil infiltration. The reason may be attributed to the reduced expression of proinflammatory cytokines and chemokines with the treatment of MgIG. The hepatoprotective effect of MgIG was associated with suppression of neutrophil ROS production as well as hepatocellular oxidative stress. MgIG may play a critical role in protecting against chronic plus binge ethanol feeding-induced liver injury by regulating neutrophil activity and hepatic oxidative stress.

## 1. Introduction

The prevalence of chronic alcohol consumption has increased in the last decades in the Western world as well as in Asian countries [1]. According to the WHO report in 2011, chronic alcohol consumption resulted in approximately 2.5 million deaths each year. Among these alcoholics, almost 20% of them developed alcoholic liver disease (ALD), which was still a leading cause of liver-related morbidity and mortality worldwide. The pathogenesis of ALD was a complex process in both parenchymal and nonparenchymal cells and other cell types recruited into the liver in response to liver damage and inflammation. Hepatocytes were damaged by increased ethanol via generation of reactive oxygen species (ROS), endoplasmic reticulum (ER) stress, and mitochondrial dysfunction [2]. The damaged hepatocytes release danger-associated molecular patterns (DAMPs), together with pathogen-associated molecular patterns (PAMPs) derived from gut bacteria due to the increased permeability by ethanol, triggered liver inflammation, and recruited neutrophils into the liver [2, 3]. The accumulation of neutrophils in the liver promoted further hepatocellular injury and inflammation which was believed to be critical in the development of ALD [4, 5]. The conventional treatment of ALD such as corticosteroids or tumor necrosis factor alpha (TNF-*α*) inhibitor therapy usually causes increased chance of infections since these drugs were immune suppressive. So, it is very important to explore novel strategies for treating ALD [3].

Magnesium isoglycyrrhizinate (MgIG), a magnesium salt of 18*α*-glycyrrhizic acid stereoisomer of glycyrrhizic acid, is clinically used for the treatment of inflammatory liver diseases in China and Japan [6–8]. MgIG has been reported to have strong anti-inflammatory, antioxidant, antiviral, and hepatoprotective activities [9–11]. MgIG may inhibit LPS-induced activation of phospholipase A_2_ (PLA_2_)/arachidonic acid (AA) pathway. Treatment of MgIG suppressed the production of AA metabolites induced by LPS, such as prostaglandin E2 (PGE2), prostacyclin (PGI2), thromboxane 2 (TXB2), and leukotrienes (LTB4) in macrophages [11]. Other studies indicated that MgIG inhibits inflammatory response through blocking STAT3 pathway activation in partial hepatectomy model and ischemia/reperfusion liver injury model [9, 12]. MgIG also showed hepatoprotective effects in drug-induced liver injury [13, 14], immune-mediated liver injury [10], and fatty liver [15]. A recent report showed that MgIG could reduce lipid accumulation induced by ethanol in vitro [16]; however, whether MgIG can be used for effectively treating ALD in vivo remains unknown.

To mimic acute-on-chronic alcoholic liver injury in patients, Bertola et al. described a novel mouse chronic plus binge ethanol feeding model (NIAAA model) for ALD [17]. Briefly, mice were subjected by chronic ethanol feeding (10 d ad libitum oral feeding with the Lieber-DeCarli ethanol liquid diet) plus a single binge dose of ethanol delivered by gavage. This model reproduced the drinking behaviors of ALD patients with elevated serum levels of alanine aminotransferase (ALT), steatosis, and neutrophil infiltration in the liver and upregulated the expression of proinflammatory cytokines. In this study, we utilized this NIAAA model to investigate the protective effects and mechanism underlying the effect of MgIG on ALD.

## 2. Materials and Methods

### 2.1. Materials

MgIG powder was provided by Nanjing Zhengda Tianqing Pharmaceutical Co. Ltd., Nanjing, China. MgIG powder was dissolved in PBS for injections.

### 2.2. Animals and NIAAA Model

Adult male C57BL/6 mice weighing 20–25 g used in this study were used for ad libitum ethanol feeding, described as the chronic plus binge alcohol feeding [17]. Lieber-DeCarli ‘82 Shake and Pour control liquid diet and Lieber-DeCarli ‘82 Shake and Pour ethanol liquid diet (Bio-Serv, Frenchtown, NJ) were prepared according to the manufacturer's instruction. Mice were fed with liquid control diet for 5 days and then switched either to a liquid diet containing 5% ethanol or a control diet for 10 days. MgIG (22.5 mg/kg or 45 mg/kg) or PBS was administered i.p. every day during the 10-day liquid diet or 10-day control diet. At day 11, mice were treated with MgIG or PBS; 2 hours later, all mice were gavaged with a single dose of ethanol (5 g/kg) or isocaloric maltodextrin. All mice were sacrificed 9 hours after gavage. The experiment was carried out with the approval of the institution animal use committee.

### 2.3. Histopathologic Evaluation

Liver specimens were collected and fixed in 10% formalin and paraffin embedded, then cut into 4 *μ*m slices, and stained with hematoxylin and eosin (H&E) and immunohistochemistry for MPO, HNE, and MDA using a rabbit ABC staining kit (Vector Laboratories, Inc., Burlingame, CA) according to the manufacturer's protocol. Primary antibodies used were listed below: antimyeloperoxidase (MPO) (Biocare Medical, Concord, CA), antimalonaldehyde (MDA) (Genox, Baltimore, MD), and 4-hydroxynonenal (4-HNE) (Genox).

### 2.4. Biochemical Assays

Serum alanine aminotransferase (ALT) and aspartate aminotransferase (AST) levels were analysed using a Catalyst Dx Chemistry Analyzer (IDEXX Laboratories, Inc., Westbrook, ME).

### 2.5. Isolation of Hepatic Total Lymphocytes

The isolation of total hepatic lymphocytes was performed as described previously [11]. In brief, mouse livers were removed and pressed through a 70 *μ*m cell strainer. The liver cell suspension was collected and suspended in PBS, followed by centrifugation at 50 ×g for 5 min. Supernatants containing total lymphocytes were collected. The pellets were resuspended in 40% Percoll in PBS and centrifuged for 15 min at 750 ×g. 3 ml ACK Lysing Buffer was added to the tubes to lyse the residual RBCs. Then, the liver lymphocytes were washed twice with PBS and resuspended in PBS with 1% fetal bovine serum in PBS for flow cytometric analysis.

### 2.6. Flow Cytometry Analysis for Neutrophils

Liver lymphocytes were stained for Gr-1, CD11b, and CD62L (eBioscience, San Diego, CA, USA). Stained cells were analyzed on Cytoflex flow cytometer (Beckman Coulter, Brea, CA).

### 2.7. Flow Cytometric Analysis of Intracellular Reactive Oxygen Species (ROS) Production

A dihydrorhodamine 123 (DHR 123) oxidation stress assay was performed as described previously [18]. Briefly, liver lymphocytes (1 × 10^6^) were incubated in 1 ml DMEM medium with 100 *μ*M DHR and 1000 U/ml catalase in 37°C for 5 minutes, and 200 ng PMA was added into medium and incubated for an additional 20 minutes. Cells were washed and resuspended in PBS for flow cytometric analysis.

### 2.8. Real-Time Quantitative Polymerase Chain Reaction (Real-Time PCR)

Total liver RNA was extracted by using the TRIzol reagent (Invitrogen, Carlsbad, CA), followed by reverse transcription into cDNA using the High-Capacity cDNA Reverse Transcription Kit (Applied Biosystems, Foster City, CA). Real-time PCR was performed using the ABI PRISM 7500 Real-Time PCR System and SYBR Green Master Mix (Applied Biosystems) according to the manufacturer's instructions. Mouse primer sequences used are shown below:

CXCL1: Forward: TCTCCGTTACTTGGGGACAC; Reverse: CCACACTCAAGAATGGTCGC.CXCL2: Forward: TCCAGGTCAGTTAGCCTTGC; Reverse: CGGTCAAAAAGTTTGCCTTG. E-Selectin: Forward: TCTATTTCCCACGATGCATTT; Reverse: CTGCCAAAGCCTTCAATCAT. IL-6: Forward: ACCAGAGGAAATTTTCAATAGGC; Reverse: TGATGCACTTGCAGAAAACA.

TNF*α*: Forward: AGGGTCTGGGCCATAGAACT; Reverse: CCACCACGCTCTTCTGTCTAC.IL-1*β*: Forward: GGTCAAAGGTTTGGAAGCAG Reverse: TGTGAAATGCCACCTTTTGA. CD14: Forward: CAGAAGCAACAGCAACAAGC; Reverse: ACTGAAGCTTTTCTCGGAGC. 18s RNA: Forward: GGCCCTGTAATTGGAATGAGTC; Reverse: CCAAGATCCAACTACGAGCTT.

### 2.9. Statistical Analyses

All data are expressed as means ± SEM. Differences among groups were analyzed by one-way analysis of variance (ANOVA), and the post hoc Student-Newman-Keuls (SNK) method was used for multiple comparisons. The *p* value reported was two sided, and a value of *p* < 0.05 was considered statistically significant. All analyses were performed using the SPSS software (Version 12.0, SPSS Inc., USA).

## 3. Results

### 3.1. Treatment of MgIG Protected Mice from Chronic Plus Binge Ethanol Feeding-Induced Liver Injury and Steatosis

**Figure 1:**
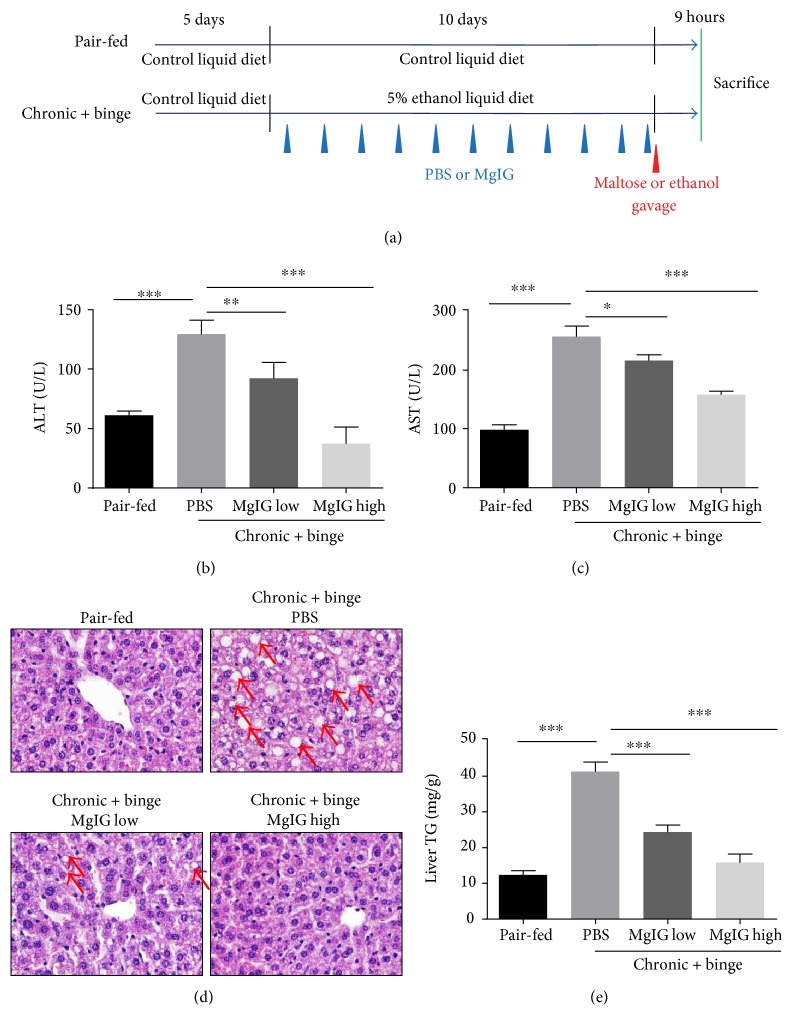
Protective effect of MgIG treatment against chronic plus binge ethanol feeding-induced hepatic injury. (a) Experimental design of liquid control or ethanol diet feeding and drug treatments. Mice were treated as described in (a); liver injury was assessed by measuring serum alanine aminotransferase (ALT) levels (b) and aspartate aminotransferase (AST) levels (c). (d) Representative H&E staining. Arrows indicate macrovesicular and microvesicular steatosis. (e) Hepatic triglyceride (TG) levels were measured. Values represent means ± SEM. ^∗^*p* < 0.05; ^∗∗^*p* < 0.01; ^∗∗∗^*p* < 0.001.

To investigate the potential hepatoprotective effects of MgIG in ALD, we used NIAAA model which can mimic major features of early ALD patients such steatosis, liver injury, and inflammation [17, 19]. MgIG was given to mice by i.p. injections daily at 22.5 mg/kg or 45 mg/kg during the course of Lieber-DeCarli ethanol liquid diet feeding and 2 hours before last ethanol gavage (Figure 1(a)). As shown in Figures 1(b) and 1(c), MgIG treatment significantly attenuated the elevation of serum ALT and AST levels induced by chronic plus binge ethanol feeding in a dose-dependent manner. It indicated that MgIG protected liver from injury caused by ethanol. In addition, MgIG greatly improved histopathological signs caused by ethanol, such as ballooning of hepatocytes and microvesicular steatosis (Figure 1(d)). Consistently, we observed a significant reduction of liver triglyceride levels in MgIG-treated mice compared with the control mice (Figure 1(e)).

### 3.2. Treatment of MgIG Blocked Chronic Plus Binge Ethanol Feeding-Induced Neutrophil Infiltration and Activation in the Liver

**Figure 2:**
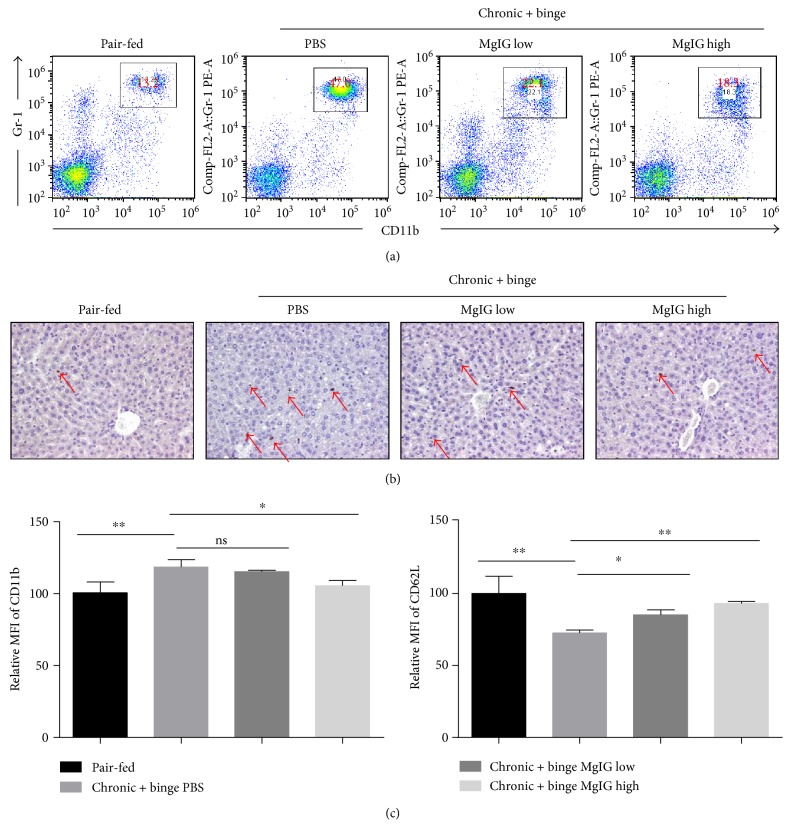
MgIG prevents chronic plus binge ethanol feeding-induced neutrophil infiltration in the liver. Mice were treated as described in Figure 1. (a) Liver leucocytes were isolated and analyzed by flow cytometry. The percentage of neutrophils (Gr1 + CD11b+) in liver leucocytes was determined. (b) Immunohistochemical staining of MPO-positive neutrophils in the liver. (c) Relative mean fluorescence intensity (MFI) of the cell surface levels of CD11b and CD62L on liver neutrophils determined by flow cytometry. Increase of CD11b and decrease of CD62L are associated with the activation of neutrophils. Values represent means ± SEM. ^∗^*p* < 0.05, ^∗∗^*p* < 0.01.

The presence of neutrophils in the liver parenchyma was a key feature of alcoholic hepatitis [5]. The infiltration of neutrophils played critical roles in the development of alcohol-induced liver damage [4, 20]. We analyzed liver neutrophils in the liver by flow cytometry. Our data confirmed that the percentages and total number of neutrophils greatly increased in the livers of chronic-binge-fed mice than in pair-fed mice in a previous report [20]. The treatment of MgIG significantly blocked the increase of both percentage of neutrophils in the liver leucocytes (Figure 2(a)). Moreover, the immunohistochemical staining of neutrophil marker myeloperoxidase (MPO) also indicated a reduction of liver neutrophils with MgIG treatment, which was consistent with the flow cytometry data (Figure 2(b)). In addition, we compared neutrophil activation marker expression by flow cytometric analysis. MgIG treatments prevent the increase of CD11b expression and the decrease of CD62L expression (Figure 2(c)), which suggested that MgIG could inhibit the activation of neutrophils in our chronic plus binge ethanol feeding model.

### 3.3. Treatment of MgIG Prevented Inflammatory Cytokine and Chemokine Production

**Figure 3:**
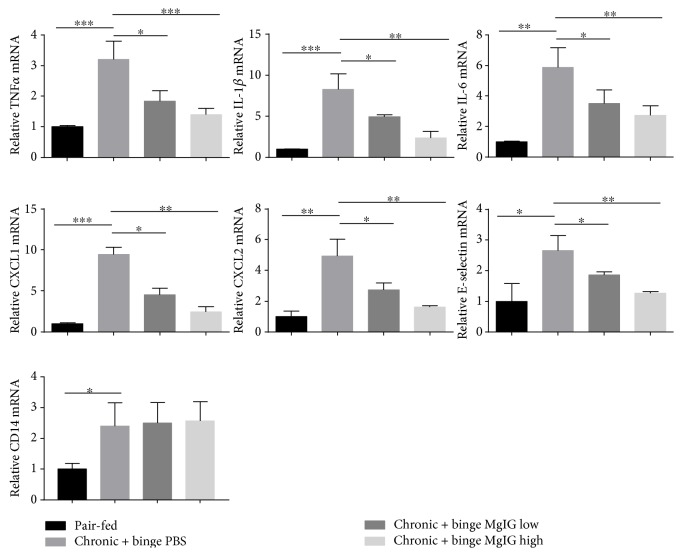
MgIG prevents chronic plus binge ethanol feeding-induced proinflammatory mediator expression. Gene expression of proinflammatory cytokines (TNF*α*, IL-1*β*, and IL-6), neutrophil migration-related chemokines (CXCL1 and CXCL2), neutrophil adhesion molecule (E-selection), and Kupffer cell activation marker (CD14) in the liver. Values represent means ± SEM. ^∗^*p* < 0.05; ^∗∗^*p* < 0.01; ^∗∗∗^*p* < 0.001.

To explore the mechanism on how MgIG prevented the infiltration and activation of neutrophils in the liver in the chronic plus binge ethanol feeding model, we measured several cytokines and chemokines related to the migration and activation of neutrophils in the liver with ALD. As shown in Figure 3, the mRNA expression levels of proinflammatory cytokines such as IL-6, IL-1*β*, and TNF-*α* greatly increased in the livers of chronic-binge-fed mice than in pair-fed mice. The treatment of MgIG dose dependently blocked the elevation of these cytokines. Similarly, the increase of chemokines for neutrophil migration CXCL1 and CXCL2 and adhesion molecule E-selectin was also blocked by MgIG treatment. To determine whether MgIG affected the initial response of Kupffer cells to LPS release from gut bacterial, we checked CD14 expression in the liver, as shown in Figure 3. CD14 expression significantly increased in chronic plus binge ethanol feeding mice; however, MgIG did not influence the elevation of CD14 in the liver.

### 3.4. MgIG Blocked Chronic Plus Binge Ethanol Feeding-Induced Neutrophil ROS Production and Oxidative Stress in the Liver

**Figure 4:**
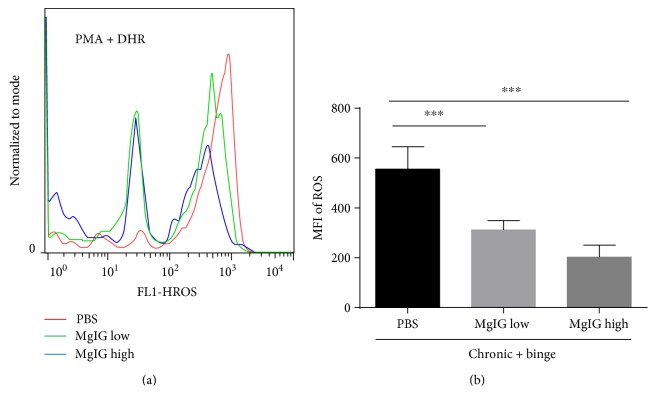
MgIG prevents chronic plus binge ethanol feeding-induced reactive oxygen species (ROS) production increase by liver neutrophils. (a) Liver neutrophils were isolated and stimulated with phorbol 12-myristate 13-acetate (PMA). ROS production was determined by dihydrorhodamine 123 (DHR 123) assay. (b) MFI of ROS was quantified. Values represent means ± SEM. ^∗∗∗^*p* < 0.001.

**Figure 5:**
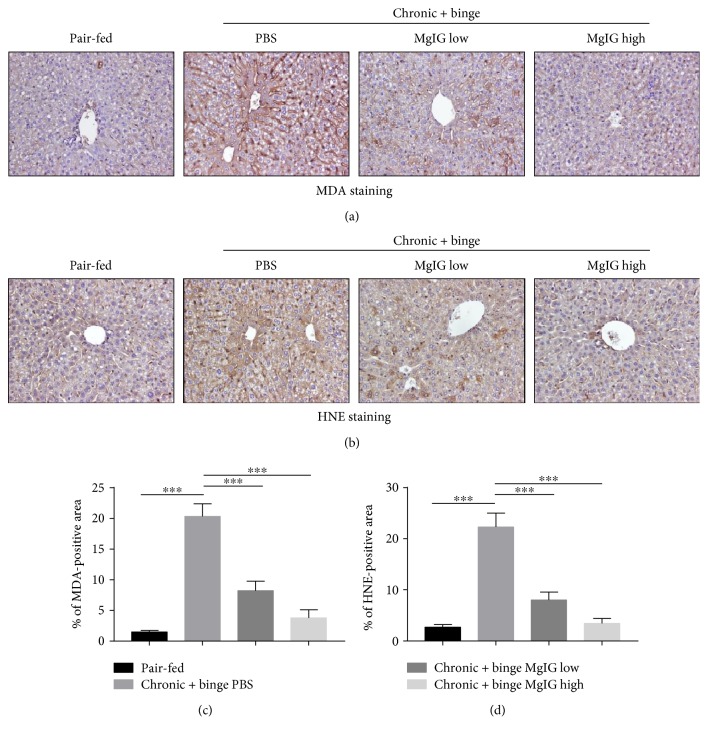
MgIG ameliorates chronic plus binge ethanol feeding-induced oxidative stress in the liver. Liver tissues were subjected to immunostaining with (a) an antimalonaldehyde (MDA) or (b) anti-4-hydroxynonenal (HNE) antibody. (c and d) Quantification of (a) and (b). Values represent means ± SEM. ^∗∗∗^*p* < 0.001.

A recent study suggested that neutrophil-derived ROS and oxidative stress played important roles in alcohol-induced liver injury [18, 21]. We checked liver neutrophil ROS production by flow cytometer. As shown in Figures 4(a) and 4(b), the treatment of MgIG significantly reduced the phorbol 12-myristate 13-acetate- (PMA-) stimulated ROS levels in hepatic neutrophils. Moreover, hepatic levels of lipid peroxide including malonaldehyde (MDA) and 4-hydroxynonenal (4-HNE) were examined by immunohistochemistry. Figures 5(a) and 5(b) showed that levels of hepatic MDA and 4-HNE expression were elevated after chronic plus binge feeding, while MgIG significantly reduced hepatic MDA and 4-HNE levels in chronic plus binge mice.

## 4. Discussion

In our study, increased oxidative stress and neutrophil cell infiltration were observed after chronic plus binge feeding treatment. The treatment of MgIG significantly blocked the activation and infiltration of neutrophils in the chronic plus binge model. Moreover, the increased ROS generation and oxidative stress induced by ethanol were attenuated by MgIG treatment. These results suggested promising hepatoprotective effects of MgIG against ALD.

Hepatic neutrophil infiltration was considered a hall marker of alcoholic hepatitis and played critical roles in the development and progression of ALD [3, 5, 22, 23]. However, the widely used chronic Lieber-DeCarli ethanol diet feeding ALD model could only trigger very mild or no neutrophil infiltration. The recently developed chronic plus binge feeding model mimics human ALD patients drinking pattern and triggers significant liver neutrophil infiltration and liver damage. The role of neutrophils has been extensively studied by using this model. Neutrophil depletion by antibody almost completely blocked the liver injury in this model. In addition, the deficiency of E-selectin, a key adhesion molecule for neutrophil migration, greatly reduced the severity of chronic plus binge-induced liver injury [17, 20]. So, targeting neutrophil may represent an effective strategy for treating ALD. Here, we adopted the chronic plus binge feeding model to evaluate the hepatoprotective effects of MgIG on ALD and possible mechanisms involved, especially how MgIG influenced the behavior of neutrophils.

MgIG, a derivative of glycyrrhizic acid, was the extraction of the plant *Glycyrrhiza glabra*, with potential anti-inflammatory and antioxidant effects. The beneficial effects of MgIG in treating liver diseases were proven in several liver disease models including drug-induced liver damage, immune-mediated liver injury, and fatty liver. In vitro studies showed that MgIG might also reduce fat accumulation induced by ethanol [8, 10–13, 15, 24–28]. The therapeutic effect of MgIG on liver inflammatory inhibition was tested in patients with viral hepatitis, alcoholic liver disease, nonalcoholic liver disease, drug-induced liver injury, and autoimmune hepatitis by a randomized, double-blind, multi-center clinical study and prospective randomized controlled study [6]. Here, we showed that MgIG could significantly block neutrophil infiltration and activation in the chronic plus binge model. The suppression of cytokine and chemokine production in the liver was observed in MgIG-treated chronic plus binge model mice. Moreover, the production of ROS in neutrophils and liver oxidative stress was also reduced with MgIG treatment in chronic plus binge model mice. Of note, neutrophil-derived ROS has been described critical in tissue damage. So, our results supported that MgIG reduced ROS production induced by ethanol and oxidative stress in the liver. As a consequence, liver injury and subsequent liver inflammation were reduced, so that the further recruitment of neutrophils was blocked.

In summary, this study demonstrated markedly hepatoprotective effects of MgIG against chronic binge ethanol-induced liver injury. The beneficial effects may attribute reduced neutrophil ROS production, hepatic oxidative stress, and proinflammatory cytokine production. The effects of MgIG in treating ALD patients need to be evaluated in the future.
